# Novel Arginine- and Proline-Rich Candidacidal Peptides Obtained through a Bioinformatic Approach

**DOI:** 10.3390/antibiotics12030472

**Published:** 2023-02-26

**Authors:** Tecla Ciociola, Laura Giovati, Tiziano De Simone, Greta Bergamaschi, Alessandro Gori, Valerio Consalvi, Stefania Conti, Alberto Vitali

**Affiliations:** 1 Department of Medicine and Surgery, University of Parma, 43126 Parma, Italy; 2 National Research Council of Italy, Istituto di Scienze e Tecnologie Chimiche (SCITEC-CNR), 20131 Milan, Italy; 3 Dipartimento di Scienze Biochimiche “A. Rossi Fanelli”, Sapienza University of Rome, 00185 Roma, Italy; 4 National Research Council of Italy, Istituto di Scienze e Tecnologie Chimiche (SCITEC-CNR), 00168 Rome, Italy

**Keywords:** antimicrobial peptides, bioinformatic analysis, *Candida* species, circular dichroism, confocal laser scanning microscopy, fungal biofilms

## Abstract

Antimicrobial resistance is a major public health concern worldwide. Albeit to a lesser extent than bacteria, fungi are also becoming increasingly resistant to antifungal drugs. Moreover, due to the small number of antifungal classes, therapy options are limited, complicating the clinical management of mycoses. In this view, antimicrobial peptides (AMPs) are a potential alternative to conventional drugs. Among these, Proline-rich antimicrobial peptides (PrAMPs), almost exclusively of animal origins, are of particular interest due to their peculiar mode of action. In this study, a search for new arginine- and proline-rich peptides from plants has been carried out with a bioinformatic approach by sequence alignment and antimicrobial prediction tools. Two peptide candidates were tested against planktonic cells and biofilms of *Candida albicans* and *Candida glabrata* strains, including resistant isolates. These peptides showed similar potent activity, with half-maximal effective concentration values in the micromolar range. In addition, some structural and functional features, revealing peculiar mechanistic behaviors, were investigated.

## 1. Introduction

In recent years, fungal pathogens have had a serious impact on global health [1,2]. The incidence of deep-seated mycoses increased along with the increase in the number of subjects at risk, such as elderly people and premature babies, immunocompromised individuals, transplant recipients, and cancer patients [3]. Invasive fungal infections are still associated with high mortality rates, particularly because of the lack of a timely diagnosis and subsequent early antifungal treatment initiation [4]. The emergence of antifungal resistance is another major problem which significantly reduces the therapeutic efficacy of available antifungal agents [5,6,7]. Furthermore, the ability of fungal cells to form biofilms has been associated with high rates of morbidity and mortality, as well as with severe drug resistance [8,9]. Species belonging to the genus *Candida* are mainly responsible for fungal infections; *C. albicans*, in particular, remains the most common etiologic agent of systemic candidiasis [10].

In the search for novel compounds for counteracting the acquisition of drug resistance, antimicrobial peptides (AMPs) are gaining increasing interest as an alternative or complement to currently available antimicrobial agents [11]. The development of AMPs as therapeutic drugs will benefit in the coming years from advancements in synthesis and delivery technologies [12].

AMPs may exhibit a broad-spectrum activity against microbial and viral pathogens [13]. In fact, the mode of action exhibited by many AMPs could be applied for different pathogens. For instance, typical AMPs, acting through cell membrane lysis, could be active on bacterial, fungal, or even cancer cells [13,14]. Many AMPs, however, are non-lytic and act inside the target pathogens by various modes of action [15,16]. Proline-rich antimicrobial peptides (PrAMPs), in particular, act by targeting intracellular pathways [17,18].

Antifungal peptides may act in different ways: as antibacterial peptides, they may cause membrane permeabilization, forming pores with the passage of ions and other solutes, and subsequent cell death. Other antifungal peptides enter the target cells and interact with intracellular molecular targets; some may interfere with cell wall synthesis, glucans, or chitin biosynthesis [19,20,21]. In addition to direct antifungal activity, some peptides can prevent biofilm formation and/or eradicate preformed biofilms by inhibiting fungal adhesion, causing cell wall perturbation, generating reactive oxygen species, and regulating gene expression [22,23,24].

Since ancient times, plants have been a source of natural remedies against diseases and, today, researchers still mine plant bioactive compounds in the search for novel drugs. A large number of plant AMPs are known, including cyclotides, defensins, knottins, snakins, and thionins, classified into different groups based on their sequence similarity and tertiary structure [25,26,27]. While many plant AMPs are rich in cysteine residues [28], to date, only one proline-rich AMP has been found in plants, the peptide BnPRP1 from *Brassica napus* [29]. 

This study focused on the search in the plant kingdom for new antimicrobial peptides rich in proline and arginine residues, as found in many AMPs of animal origin [18]. For this purpose, we used bioinformatic tools to find, in the plant proteome database, proteins containing selected sequence stretches to be further evaluated for the prediction of antifungal activity. This search resulted in two peptides, named Ma1 and Ma2. Both were shown to possess potent anticandidal activity against planktonic and biofilm-structured *Candida* cells. Different from many PrAMPs from animals, their mode of action appeared to be linked to a membrane perturbation mechanism, as suggested by the killing kinetics assay, the in vitro vesicles perturbation assay, and confocal microscopy. Structural features have been assessed with circular dichroism measurements, suggesting a predominant random coil arrangement.

## 2. Results

### 2.1. Bioinformatic Analysis

**Scheme 1:**
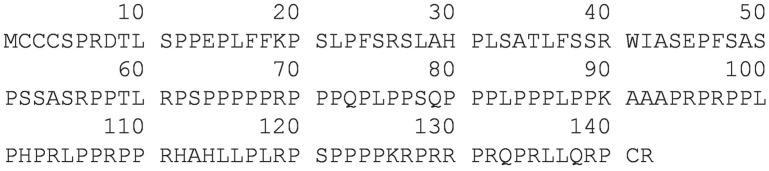
Sequence of the M0T9Q1 (M0T9Q1_MUSAM) uncharacterized protein, now coded as UPI000295EFC0 in the UniParc database.

To individuate proline- and arginine-rich motifs in plant proteins using the BLAST search engine within the plants database, we arbitrarily chose, as a template, the fused sequences PRPPYLPRPRP and PRIPPG, corresponding to the 4–14 and 24–29 residues of the antimicrobial peptide PR-39 isolated from pig intestine [30]. At the time of the search, the best match was found for an uncharacterized protein of *Musa acuminata* (M0T9Q1_MUSAM), whose sequence is reported in Scheme 1.

Currently, this sequence is no longer available in UniProtKB, as the protein file was deleted and stored in the UniParc database with the code UPI000295EFC0. Different proline- and arginine-rich motifs were found, especially in the 94–140 residues frame.

From this frame, 15 adjacent residues stretches were selected and submitted to in silico predictive analyses for antifungal activity employing the Deep-AFPpred (https://afppred.anvil.app/) (accessed on 7 February 2022), a recent deep-learning-based server specifically designed to predict antifungal propensity in peptides [31].

The two sequences PPKRPRRPRQPRLLQ and KRPRRPRQPRLLQRP, among others, showed high scores (0.9993 and 0.9996, respectively). The two peptides were named Ma1 and Ma2 and were synthesized for further studies.

### 2.2. Structural Studies on Ma1 and Ma2

**Figure 1:**
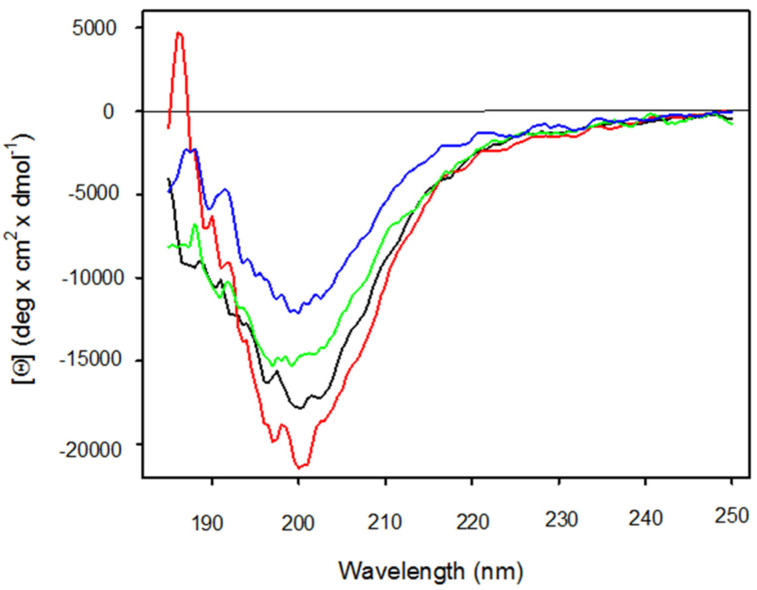
Far-UV CD spectra of peptides in water (Ma1 black line, Ma2 green line) and in 30% TFE (Ma1 red line, Ma2 blue line).

Ma1 and Ma2 peptides have been analyzed by circular dichroism (CD) spectroscopy; far-UV spectra were recorded in the range of 185–250 nm. Ma1 and Ma2, in water, showed spectra characterized by a deep negative band around 200 nm (π–π∗), which were slightly less pronounced for the peptide Ma2 (Figure 1). This band can be, in general, assigned to a periodic unordered secondary structure conformation. Despite the presence of several proline residues in the Ma1 and Ma2 sequences, the lack of the typical weak positive band around 220 nm excludes the presence of a polyPro II conformation for both peptides [32,33]. The addition of 30% 2,2,2-trifluorethanol (TFE) did not change the shape of the peptides’ spectra but rather the intensity of their signals. The spectrum of Ma1 showed a modest increase in the negative values of ellipticity; conversely, that of Ma2 showed a decrease in negative ellipticity. In the case of the peptide Ma1 spectrum, a zero intercept at around 190 nm was observed when 30% TFE was added.

### 2.3. Activity of Peptides against Planktonic Yeast Cells

The Ma1 and Ma2 peptides were proven to have direct candidacidal activity against all the investigated strains, as assessed by colony forming unit (CFU) assays, with values of half-maximal effective concentration (EC_50_) ranging between 0.108 and 0.416 µM (Table 1). Both peptides showed the highest activity against the *C. albicans* SC5314 strain.

**Figure 2:**
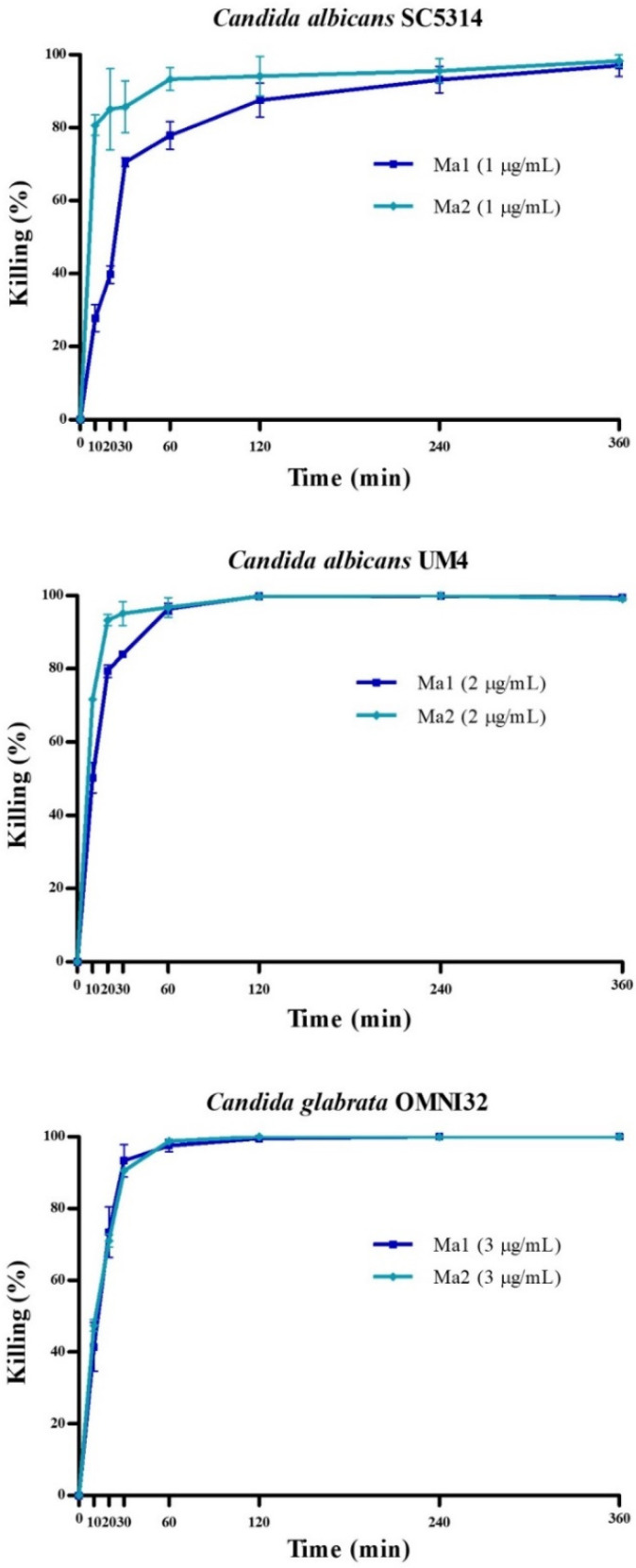
Time kinetics of in vitro activity of Ma1 and Ma2 against *Candida albicans* SC5314, *C. albicans* UM4, and *C. glabrata* OMNI32. The minimal fungicidal concentrations were used for both peptides. Reported data represent mean values ± standard deviation.

The rates of *Candida* killing by Ma1 and Ma2 over time are shown in Figure 2.

The candidacidal activity of peptides was very fast against all the investigated strains. After 30 min, in fact, the killing ranged from 70.50% (*C. albicans* SC5314) to 93.34% (*C. glabrata* OMNI32) for the Ma1 peptide and from 85.71% (*C. albicans* SC5314) to 95.12% (*C. albicans* UM4) for the Ma2 peptide. Ma2 showed a faster killing than Ma1, except for *C. glabrata* OMNI32 (93.34% and 90.51% after 30 min, respectively).

The induction of apoptosis in *Candida* cells, following the exposure to Ma1 and Ma2, was investigated by flow cytometry, exploiting the detection of phosphatidylserine on the cell surface. Under the experimental conditions adopted, the peptides were not able to induce apoptosis in all the investigated strains (Figure S1).

### 2.4. Activity of Peptides against Candida Strains’ Monomicrobial Biofilm

The crystal violet (CV) assay was used for microbial biomass quantification to evaluate the capability of Ma1 and Ma2 to reduce a monomicrobial biofilm formed by the investigated *Candida* strains in polystyrene multi-well plates.

At the higher concentration used (50 μg/mL), Ma1 and Ma2 reduced *Candida* early-stage biofilms by more than 99%. The obtained EC_50_ values are shown in Table 2. Ma2 showed a higher activity than Ma1 against all the investigated strains. *C. albicans* UM4 was the most sensitive strain to the action of both peptides, with EC_50_ values of 0.880 µmol/L and 0.229 µmol/L for Ma1 and Ma2, respectively.

Apart from a negligible effect of Ma1 against *C. albicans* SC5314 (11.64% biomass reduction), both peptides reduced the mature *Candida* biofilm biomass. Ma2 was more active than Ma1, except against *C. albicans* UM4, with a biomass reduction higher than 54% for all the tested strains. The highest activity was observed for Ma1 against *C. albicans* UM4 and for Ma2 against *C. glabrata* OMNI32, with a biomass reduction of 72.19% and 71.92%, respectively (Table 3).

### 2.5. Confocal Microscopy Studies

**Figure 3:**
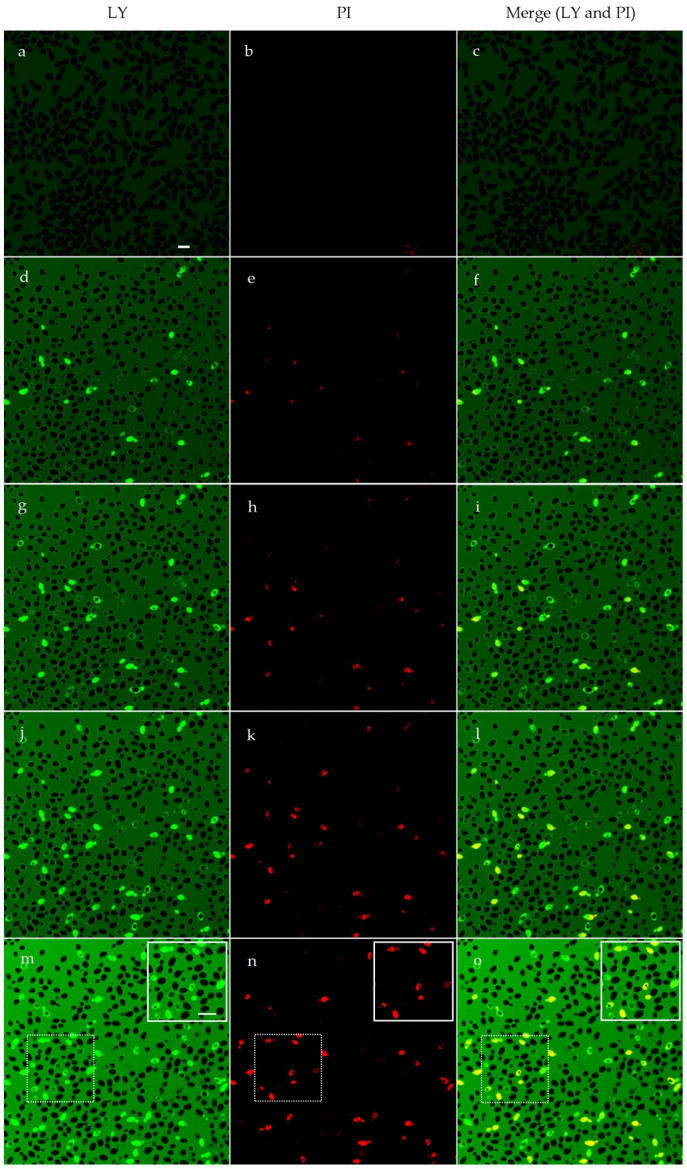
Confocal microscopy images of living *Candida albicans* SC5314 cells treated with Ma1. (**a**–**c**) Yeast cells, pre-treated with propidium iodide (PI) and lucifer yellow (LY), were treated with Ma1. Images were taken after (**d**–**f**) 10 min, (**g**–**i**) 20 min, (**j**–**l**) 30 min, and (**m**–**o**) 60 min. The same field is shown. Over time, LY is increasingly evidenced inside yeast cells, with subsequent internalization of PI, suggesting an irreversible membrane permeabilization induced by the peptide, eventually resulting in cell death. Prior to internalization, LY is localized at the cell wall level. The inset in panels (**m**–**o**) shows a detail of the field highlighted by dotting. Bar, 10 μm.

**Figure 4:**
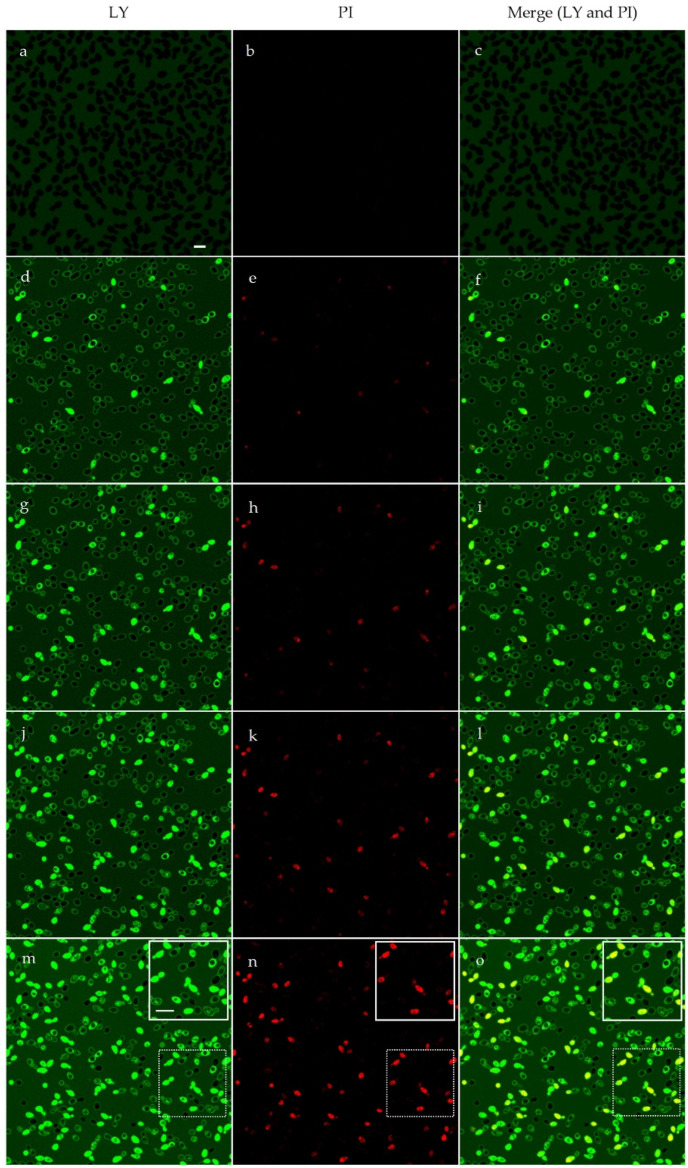
Confocal microscopy images of living *Candida albicans* SC5314 cells treated with Ma2. (**a**–**c**) Yeast cells, pre-treated with propidium iodide (PI) and lucifer yellow (LY), were treated with Ma2. Images were taken after (**d**–**f**) 10 min, (**g**–**i**) 20 min, (**j**–**l**) 30 min, and (**m**–**o**) 60 min. The same field is shown. Over time, LY is increasingly evidenced inside yeast cells, with subsequent internalization of PI, suggesting an irreversible membrane permeabilization induced by the peptide, eventually resulting in cell death. Prior to internalization, LY is localized at the cell wall level. The inset in panels (**m**–**o**) shows a detail of the field highlighted by dotting. Bar, 10 μm.

Time-lapse confocal microscopy allowed for the investigation of membrane permeabilization, following peptide treatment, in living *Candida* cells. An irreversible membrane permeabilization was observed starting after 10 min of treatment with Ma1 and Ma2 in all the investigated strains, as demonstrated by the internalization of lucifer yellow (LY) and propidium iodide (PI) in yeast cells. Images of *C. albicans* SC5314 cells treated with Ma1 and Ma2 are shown in Figure 3 and Figure 4, respectively. In the adopted experimental conditions, the candidacidal activity of peptides after 30 min of treatment was 46.42% for Ma1 and 44.95% for Ma2, as assessed by a CFU assay. The effects of Ma1 and Ma2 treatment on *C. albicans* UM4 and *C. glabrata* OMNI32 cells are reported in Figures S2–S5.

### 2.6. Artificial Vesicle Assay

**Figure 5:**
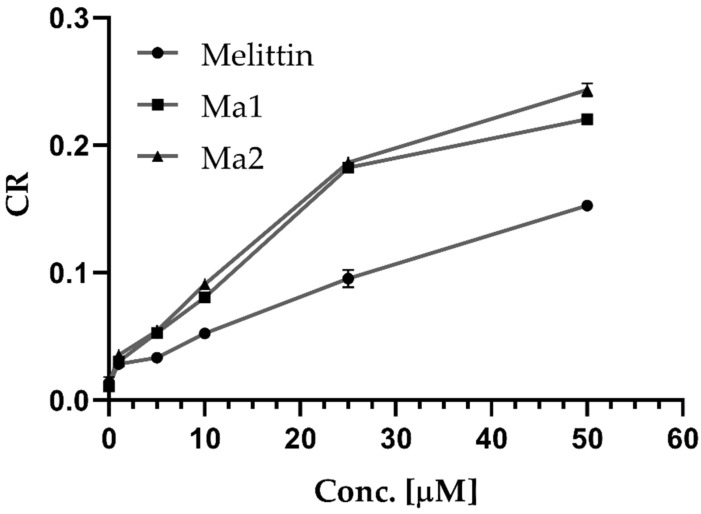
Lipid vesicle assay. PC/PDA vesicles were treated with different amounts of Ma1, Ma2, and melittin as a positive control. Data are reported as the mean ± SD.

To confirm the effect of Ma1 and Ma2 on cell membranes, a lipid vesicle assay was employed. Phosphatidylcholine (PC)/10,12-Tricosadiynoic acid (polydiacetylene, PDA) lipid vesicles were treated with Ma1 and Ma2, using melittin, a known pore-forming peptide, as a positive standard [34]. After 20 min of contact with the peptides, the vesicles turned their color from blue to pink or red, depending on the intensity of the effect. Both peptides showed a strong perturbative effect towards lipid vesicles, more so than melittin did, as a positive control. In Figure 5, the curves based on the values of colorimetric response (CR) are shown, calculated as reported in the Materials and Methods section.

### 2.7. Cytotoxicity of Peptides

**Figure 6:**
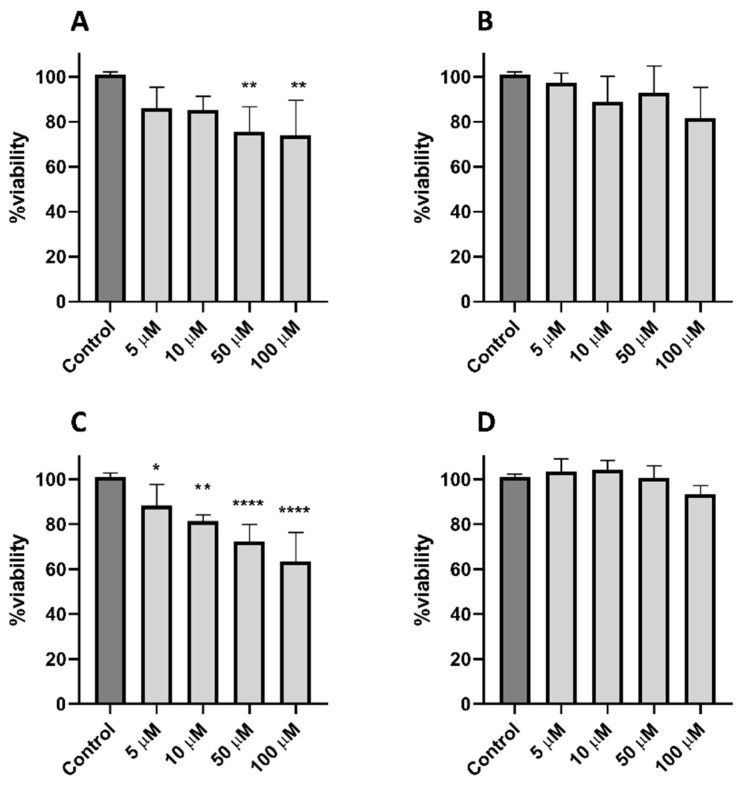
Cytotoxicity tests performed on 3T3 murine fibroblasts with Ma1 and Ma2 peptides. (**A**) Ma1 at 24 h, (**B**) Ma2 at 24 h, (**C**) Ma1 at 48 h, and (**D**) Ma2 at 48 h. All data are represented as the mean ± SD, and one-way ANOVA with Dunnett’s multiple comparisons test was performed in comparison with the Control (* *p* < 0.05, ** *p* < 0.01, **** *p* < 0.0001).

To establish potential cytotoxic effects, Ma1 and Ma2 were tested on the murine 3T3 fibroblast cell line. The two peptides showed a slight increasing cytotoxicity, conforming to the increasing concentrations of the two peptides, as evidenced by a colorimetric cell viability assay (Figure 6).

## 3. Discussion

The identification of new antimicrobial molecules is urgently needed to counteract the ever-growing phenomenon of antimicrobial resistance. In this study, two peptides have been identified as novel potential antifungal molecules.

The original rationale at the basis of our search in the plant proteomes was to identify sequences rich in proline and arginine residues, which are typically found in PrAMPs in animals [18], with the exception of BnPRP1, the first reported proline-rich antimicrobial peptide in plants [29]. We set our search through BLAST using the PRPPYLPRPRPPRIPPG sequence as a template, which was the result of the arbitrary fusion of stretches (4–14; 24–29) derived from the pig antimicrobial peptide PR-39 [30], chosen as a reference. At the time of our search, an uncharacterized protein from *Musa acuminata* coded as M0T9Q1_MUSAM gave the best result in terms of the similarity with the arbitrary sequence that we used as a template. In September 2021, the M0TQ1 protein was deleted from the UniProtKB database (now stored in the UniParc database with the code UPI000295EFC0), leaving the obtained sequences without a real protein reference.

When a predictive search was carried out to find potential antifungal stretches within the protein by using the recent deep-learning-based prediction Deep-AFPpred server [31], among the sequences with higher scores, two peptides, named Ma1 and Ma2, were selected. The number of studies concerning the use of prediction sites for the discovery of new antimicrobial peptides is growing, thanks to an increasing prediction accuracy derived from continuously updated datasets and to the use of artificial intelligence-based algorithms, allowing for the potential of these sites to be expanded [35,36]. The data obtained from the antifungal assays for Ma1 and Ma2 are of interest because the accuracy and goodness of the prediction made by the Deep-AFPpred server were experimentally confirmed.

Ma1 and Ma2 have shown strong antifungal activity at micromolar concentrations against all the investigated *Candida* strains, including drug-resistant isolates, both planktonic and organized in biofilm. Generally, Ma2 was more effective than Ma1 under the adopted experimental conditions, and *C. albicans* UM4, when organized in biofilm, was the strain more sensitive to the action of both peptides.

From a functional point of view, the activity of Ma1 and Ma2 presents some interesting aspects. Usually, proline/arginine-rich AMPs express their mechanism of action by interacting with intracellular targets such as ribosomes or DnaK and GroEL chaperonins or by interfering with the translational process [35], while a membranolytic or perturbative effect is less described for these families of AMPs. To our knowledge, an exception in the proline/arginine-rich antimicrobial peptides panorama is represented by the caprine bactenecin ChBac3.4, which shows a perturbative effect on bacterial membranes [36]. Other examples are from peptides employed at concentrations well above the MIC, as described for Arasin-1 [37] and Bac7 [38].

The structural investigation, performed on Ma1 and Ma2 via circular dichroism, suggests the assumption for the two peptides of a random coil structure in both water and 30% TFE conditions, as observed in other peptides containing a remarkable number of proline residues [39].

Interestingly, the lack of propensity to assume an α-helical structure even in TFE 30% hardly fits with the functional behavior of Ma1 and Ma2. In fact, these peptides were able to disrupt artificial lipid vesicles more potently than melittin, a typical α-helical peptide, showed fast killing kinetics, and induced membrane permeabilization, as evidenced by time-lapse confocal images. From a mechanistic point of view, the slight differences in the antifungal activities displayed by Ma1 and Ma2 may be based on chemical and physical characteristics, such as the charge, total hydrophobicity, and isoelectric points (calculated by the PepDraw tool, https://pepdraw.com/) (accessed on 7 February 2022). Ma2 shows a higher basic character (charge +7) with respect to Ma1 (charge +6), while the isoelectric points (p.I.) and hydrophobic characters are very similar (theoretical p.I.: 13.09 and 13.18, +19.49 Kcal · mol^−1^ and +21.16 Kcal · mol^−1^, respectively). In this view, a higher basic character of Ma2 may promote a better interaction with the target membranes, thus explaining the higher overall activity of this peptide. Further investigations will be carried out in order to elucidate the membrane/peptides interaction.

Despite the high sequence similarity, the cytotoxic effect on mammalian cells was especially evident for Ma1 and evident to a lesser extent for Ma2; statistical significance was only reached for Ma1 at concentrations ≥50 μM at 24 h or at 48 h. However, the cytotoxic effect was expressed at concentrations well above the EC_50_ calculated for this peptide.

## 4. Materials and Methods

### 4.1. Chemicals and Reagents

All of the N-α-Fmoc-L-amino acids and chlorotrityl chloride resin (CTC) used for the peptide synthesis were purchased from Iris Biotech GmbH (Marktredwitz, Germany). Fmoc-PEG building block, 8-(9-Fluorenyloxycarbonyl-amino)-3,6-dioxaoctanoic acid (Fmoc-O2Oc-OH), oxyma pure, and diisopropyl carbodiimide (DIC) were also from Iris Biotech. Trifluoroacetic acid (TFA) and N,N′-dimethylformamide (DMF) were from Carlo Erba (Rodano, Italy). Dichloromethane (DCM), N,N′-diisopropylethylamine (DIEA), phosphatidylcholine (PC), 10,12-tricosadiynoic acid (polydiacetylene, PDA), and all other organic reagents and solvents were purchased in high purity from Merck KGaA (Darmstadt, Germany), unless stated otherwise. All of the solvents for the solid-phase peptide synthesis (SPPS) were used without further purification. For the preparation of all of the solvents for liquid chromatography, HPLC-grade acetonitrile and ultrapure 18.2 Ω water (MilliQ) were used.

### 4.2. Bioinformatic Analyses

The search for proline- and arginine-rich sequences was carried out using the BLAST algorithm on the UniProt website (https://www.uniprot.org/blast/) (accessed on 1 June 2019), selecting the plants database and using the PRPPYLPRPRPPRIPPG sequence as a template. The Deep-AFPpred website (https://afppred.anvil.app/) (accessed on 7 February 2022), whose dataset consists of antifungal peptides, was used to predict the antifungal properties of selected sequences. The Deep-AFPred values of accuracy, sensitivity, precision, F1 score, specificity, and Area Under Receiver Operating Characteristic curve (AUROC) are, respectively: 94.27, 92.72, 95.69, 94.18, 95.82, and 97.84 [31].

### 4.3. Peptide Synthesis and Reversed-Phase High-Performance Liquid Chromatography (RP-HPLC) Analysis and Purification

Peptide synthesis and purification were performed as previously described, with some modifications [40]. The peptides were assembled by stepwise microwave-assisted Fmoc-SPPS on a Biotage ALSTRA Initiator+ peptide synthesizer, operating on a 0.12 mmol scale on a 2-CTC resin (0.6 mmol/g). The activation and coupling of Fmoc-protected amino acids were performed using Oxyma 0.5 M/DIC 0.5 M (1:1:1) in DMF, with a five-equivalent excess over the initial resin loading. The coupling steps were performed for 10 min at 50 °C. The deprotection steps were performed by treatment with a 20% piperidine solution in DMF at room temperature (1 × 3 min + 1 × 5 min). Following each coupling or deprotection step, peptidyl resin was washed with DMF (4 × 5 mL). The peptide was cleaved from the resin using a TFA 90%, water 5%, thioanisole 2.5%, and TIS 2.5% mixture (3 h, room temperature). After precipitation in cold diethyl ether, the crude peptide was obtained by centrifugation and rinsed with additional cold diethyl ether to eliminate scavengers. Then, the peptides were dissolved in a buffer containing 50% aqueous acetonitrile and 0.07% TFA and purified by preparative RP-HPLC.

Analytical and semi-preparative RP-HPLC were carried out on a Shimadzu Prominence HPLC system (Shimadzu Italia, Milan, Italy) equipped with a multichannel detector. Phenomenex Jupiter C18 90Å columns were used for analytical runs and peptide purification (Phenomenex, Torrance, CA, USA). The data were recorded and processed with LabSolutions software. Then, 5–100% linear gradient eluent B at a flow rate of 0.5 mL/min was used for analytic purposes (20 min run). HPLC eluent A: 97.5% H_2_O, 2.5% ACN, 0.7% TFA; HPLC eluent B: 30% H_2_O, 70% ACN, 0.7% TFA. UV detection was recorded in the 220–320 nm range. The peptides were purified by preparative RP-HPLC at a flow rate of 14 mL/min using a 100% A to 30% B gradient for 40 min. Pure RP-HPLC fractions (>95%) were combined and lyophilized. The HPLC chromatograms and the mass spectra of the final products are presented in Figures S6 and S7 and in Table S1.

### 4.4. Circular Dichroism (CD) Measurement

Far-UV CD (185–250 nm) measurements were performed at 20 °C in 0.01 cm path length quartz cuvettes with detachable windows on 1 mg of each compound dissolved in ultra-high-quality water, in the presence or absence of 30% TFE. CD measurements were performed with a Jasco-815 spectropolarimeter (Jasco, Easton, MD, USA), equipped with a Peltier thermal controller set at 20 °C. The results were expressed as the mean residue ellipticity [Θ].

### 4.5. Evaluation of Peptides’ Activity against Planktonic Yeast Cells

The candidacidal activity of Ma1 and Ma2 peptides was evaluated by conventional CFU assays, as previously described [41]. The peptides were tested at different concentrations, and the half-maximal effective concentration (EC_50_) was determined. The investigated fungal strains were *C. albicans* SC5314 (well-known reference laboratory strain), *C. albicans* UM4 (caspofungin-resistant), and *C. glabrata* OMNI32 (fluconazole-, itraconazole-, and voriconazole-resistant) [42]. Approximately 500 fungal cells were suspended in 100 µL of distilled water in the absence (control) or presence of peptides. After incubation for 6 h at 37 °C, the cell suspensions were plated on Sabouraud dextrose agar. The CFUs were enumerated after 48–72 h of incubation at 30 °C, and the candidacidal activity was determined as the percentage of CFU reduction. Each assay was carried out in triplicate, and for each condition at least two independent experiments were performed. EC_50_ was calculated by nonlinear regression analysis using GraphPad Prism 5 software (San Diego, CA, USA). Afterwards, the kinetics of the Ma1 and Ma2 killing activity, at the concentrations of 1, 2, and 3 µg/mL for *C. albicans* SC5314, *C. albicans* UM4, and *C. glabrata* OMNI32, respectively, corresponding to the minimal fungicidal concentrations at 6 h, were determined by CFU assays. The samples were collected for CFU determination after 10, 20, 30, 60, 120, 240, and 360 min of incubation.

### 4.6. Evaluation of Apoptosis Induction in Candida Cells after Treatment with Peptides

The induction of apoptosis in yeast cells following the treatment with Ma1 and Ma2 was evaluated as previously described [43]. *Candida* cells were suspended in water (5 × 10^5^ cells/mL) in the presence or absence (control) of peptides, at their 2× EC_50_ value, for 30, 60, and 120 min. The Muse Annexin V & Dead Cell Assay kit (Luminex Corporation, Austin, TX, USA) was used for the evaluation of the apoptotic profile. Data were acquired by the Muse Cell Analyzer (Luminex), according to the manufacturer’s instructions. For each condition, at least two independent experiments were performed. Differences between the peptides-treated groups and the control were assessed by an unpaired two-tailed t-Student test. A value of *p* < 0.05 was considered significant.

### 4.7. Biofilm Assays

To assess the effects of Ma1 and Ma2 peptides on the ability of *Candida* strains to produce biofilm, yeast cells were cultured in yeast extract, peptone, and dextrose (YPD) broth and incubated at 30 °C overnight with shaking (150 rpm). After washing, *C. albicans* SC5314, *C. albicans* UM4, and *C. glabrata* OMNI32 suspensions were standardized at a final concentration of 1, 0.2, and 5 × 10^6^ cells/mL, respectively, in RPMI-1640 Medium (Merck KGaA, Darmstadt, Germany) with 2% glucose (RPMI-G). The suspensions were transferred (100 μL/well) into 96-well polystyrene flat-bottomed microtiter plates and incubated at 37 °C. The effect of the treatment with peptides was evaluated on both the early-stage biofilms and the mature biofilms, as previously described [44].

#### 4.7.1. Peptide Treatment on the Early Stage of Biofilm Formation

To investigate the effect of peptide treatment on the early stages of biofilm formation, the yeast suspensions were incubated for 90 min at 37 °C; then, non-adherent cells were removed from each well by washing with sterile water. Adherent cells were treated with 100 μL of sterile water (control) or with 100 μL of Ma1 and Ma2 (final concentrations 1, 2, 3, 4, 5, 10, 25, and 50 µg/mL) for 6 h at 37 °C. After washing, fresh RPMI-G (100 μL/well) was added, and the plates were further incubated at 37 °C for 24 h in a wet chamber. Each assay was performed in triplicate, and at least two independent experiments were performed for each condition.

#### 4.7.2. Peptide Treatment on Mature Biofilm

To investigate the effect of peptides on mature biofilm, yeast suspensions were incubated at 37 °C for 24 h in a wet chamber; then, after washing, the biofilms were treated with 100 μL of water (control) or peptides at a final concentration of 50 µg/mL for 6 h at 37 °C. Each assay was performed in triplicate, and at least two independent experiments were performed for each condition.

#### 4.7.3. Biofilm Biomass Quantification

The biofilm biomass of the treated and control samples was quantified by a crystal violet (CV) assay. Briefly, after treatments, the wells were washed with water, fixed by air drying, stained with 100 μL of 0.25% CV (CV, Biolife, Milan, Italy) for 15 min in the dark, washed three times with water, and eluted with 85% ethanol. The biofilm biomasses were quantified by measuring the absorbance at 540 nm with a Multiskan Ascent Plate Reader (Thermo Labsystems). The results were expressed as the percentage of biofilm inhibition in the treated samples vs. the controls. EC_50_ was calculated by nonlinear regression analysis using GraphPad Prism 5 software.

### 4.8. Confocal Microscopy Studies

The potential role of Ma1 and Ma2 peptides in the membrane permeabilization of living *Candida* cells was studied by confocal microscopy. Yeast cells, grown overnight at 30 °C in YPD broth with shaking (130 rpm), were washed once with water; then, 2 × 10^7^ cells/mL were loaded with 1.5 μM PI and 500 μM LY. PI is a membrane impermeant dye that stains nonviable, while LY is a fluorescent molecule used as a marker of the cell membrane permeabilization. Cell suspensions (20 μL) were seeded on coverslips mounted in a special flow chamber, and, after 30 min, peptides were added (final concentrations 50 μM). Images were acquired with the STELLARIS 5 Confocal System (Leica Microsystems, Wetzlar, Germany), integrated with White Light laser and Diode UV Laser, combined with HyDS spectral detectors, and equipped with the DMi8 inverted microscope. Images were taken every 10 min for up to 60 min with an HC PL APO CS2 63x/1.40 (NA) OIL objective. A selected field was kept and observed during the time-lapse experiment. PI and LY were excited at 514 nm with WLL (white light laser) and at 405 nm with a UV laser, respectively. Acquisition was carried out in a sequential mode.

The candidacidal activities of the peptides under the adopted conditions were verified, as described above.

### 4.9. Artificial Vescicles Assay

With a few modifications, the phospholipid vesicles were produced as reported in [45]. Briefly, a final concentration of total lipids of 1 mM was achieved by combining PC and PDA in a 3:1 PC/PDA molar ratio. After being dissolved in a 2:1 solution of chloroform and methanol, the mixture was dried by rotary evaporation, resuspended in ultrapure water, warmed at 70 °C, and sonicated. The obtained opalescent solution was then cooled at 4 °C overnight.

Before use, the vesicle preparation was centrifuged at 2000 rpm for 15 min at 25 °C, and the suspension was subsequently polymerized under UV irradiation at 220 nm for a few seconds. The polymerization reaction was assessed by reaching an intense blue color. The vesicles were treated with different concentrations of Ma1, Ma2, and melittin as a positive control (0.5, 1, 5, 10, 25, and 50 µM) [34] for 20 min, and the colorimetric conversion (blue–red color transition) followed, reading the signals at 500 and 640 nm with a spectrophotometer (Agilent 8453, Santa Clara, CA, USA). To quantitatively evaluate the effect of peptides, the colorimetric response (%CR) was calculated as:

%CR = [(PB0 − PBI)/PB0] × 100

where PB = A_640_/(A_640_ + A_500_), A is the absorbance at 640 nm (blue color) and 500 nm (red component) in the UV–vis spectrum, PB0 is the red/blue ratio of the control sample (before the color change), and PBI is the value obtained for the vesicle solution after the addition of peptides. Each experiment was performed in triplicate.

### 4.10. Cytotoxicity Tests

To verify the cytotoxicity of Ma1 and Ma2 peptides, mouse 3T3-NIH fibroblasts were used. The cells were cultured at 37 °C in a humidified environment (5% CO_2_) using Dulbecco’s Modified Eagle’s medium (DMEM) supplemented with 10% Fetal Calf Serum, 20 mM L-glutamine, 10 mg/mL streptomycin, and 500 units/mL penicillin. In toxicity assays, cells (1 × 10^4^) in basal medium (200 µL) were seeded in a 96-well tissue culture plate. Sub-confluent cells were synchronized for 24 h in a serum-free medium. Cellular viability was evaluated using the CellTiter-Blue^®^ Cell Viability Assay (Promega, WI, USA) after 24 h and 48 h upon the addition of the peptide solutions (in PBS) at different concentrations (5, 10, 50, and 100 µM). The resulting fluorescence (560Ex/590Em) of the solution was determined using an automatic microplate reader (Glomax, Promega, WI, USA.), performing the readings after a suitable treatment time with the reagent. Each experiment was performed in triplicate. Differences between the peptides-treated groups and the control were assessed by one-way ANOVA with Dunnett’s multiple comparisons test. A value of *p* < 0.05 was considered significant.

## 5. Conclusions

Two new antifungal peptides were identified in this study through a bioinformatic search for plant proteins rich in arginine and proline residues. Ma1 and Ma2 sequences have been identified within the unknown protein UPI000295EFC0, previously referred to *Musa acuminata* (banana fruit), and predicted to be antifungal using the prediction bioinformatic tool DeepAFpred. Their activity was confirmed against planktonic cells and biofilms of *C. albicans* and *C. glabrata* strains, including resistant isolates. Interestingly, despite their physico-chemical features, they seem to exert a membrane-perturbation effect, while most proline- and arginine-rich AMPs of animal origin act on intracellular targets. Further studies are needed to shed light on this mechanistic aspect. The Ma2 peptide, in particular, showed more potent activity in the micromolar range and non-significant cytotoxicity against mammalian cells, thus representing a promising candidate for developing new antifungal drugs. For this purpose, future research will be needed to evaluate the in vivo therapeutic activity of these peptides, or their derivatives, in suitable experimental infection models (e.g., *Galleria mellonella* larvae).

## Figures and Tables

**Table 1:** In vitro anti-*Candida* activity of Ma1 and Ma2 peptides against planktonic cells.

*Candida* Strains	EC_50_ (95% Confidence Intervals) [mol/L]	
	Ma1	Ma2
*C. albicans* SC5314	1.241 (1.149–1.341) × 10^−7^	1.079 (0.959–1.214) × 10^−7^
*C. albicans* UM4	1.532 (1.462–1.605) × 10^−7^	2.038 (1.682–2.468) × 10^−7^
*C. glabrata* OMNI32	4.160 (2.541–6.812) × 10^−7^	3.907 (3.350–4.558) × 10^−7^

EC_50_, half-maximal effective concentration.

**Table 2:** In vitro activity of Ma1 and Ma2 against early-stage *Candida* biofilm.

*Candida* Strains	EC_50_ (95% Confidence Intervals) [mol/L]	
	Ma1	Ma2
*C. albicans* SC5314	2.842 (2.677–3.018) × 10^−6^	0.554 (0.373–0.823) × 10^−6^
*C. albicans* UM4	0.880 (0.809–0.956) × 10^−6^	0.229 (0.220–0.239) × 10^−6^
*C. glabrata* OMNI32	1.968 (1.504–2.577) × 10^−6^	1.181 (1.021–1.367) × 10^−6^

EC_50_, half-maximal effective concentration.

**Table 3:** In vitro activity of Ma1 and Ma2 against mature *Candida* biofilm.

*Candida* Strains	% Biomass Reduction at 50 μg/mL	
	Ma1	Ma2
*C. albicans* SC5314	11.64 ± 0.19	57.31 ± 0.79
*C. albicans* UM4	72.19 ± 0.63	54.44 ± 2.46
*C. glabrata* OMNI32	49.84 ± 0.10	71.92 ± 3.55

## Data Availability

The data summarized in this study are available on request from the corresponding author.

## Supplementary Materials

The following are available online at https://www.mdpi.com/article/10.3390/antibiotics12030472/s1, Figure S1: Apoptotic effects of the treatment with Ma1 and Ma2 in *Candida* strains. Figure S2: Confocal microscopy images of living *Candida albicans* UM4 cells treated with Ma1. Figure S3: Confocal microscopy images of living *Candida albicans* UM4 cells treated with Ma2. Figure S4: Confocal microscopy images of living *Candida glabrata* OMNI32 cells treated with Ma1. Figure S5: Confocal microscopy images of living *Candida glabrata* OMNI32 cells treated with Ma2. Figure S6: HPLC chromatogram (upper scheme) and mass spectrum (lower scheme) of the Ma1 peptide. Figure S7: HPLC chromatogram (upper scheme) and mass spectrum (lower scheme) of the Ma2 peptide. Table S1: *m*/*z* values of the obtained mass spectra of the peptides Ma1 and Ma2.
