# Supplementary material for: Novel Arginine- and Proline-Rich Candidacidal Peptides Obtained through a Bioinformatic Approach
Source: Antibiotics (Basel). 2023 Feb 26;12(3):472. doi: 10.3390/antibiotics12030472 (PMC10044544; doi:10.3390/antibiotics12030472)
Supplement: Supplementary file 1 [file antibiotics-12-00472-s001.zip › antibiotics-2213628-supplementary.pdf]

Novel arginine- and proline-rich candidacidal peptides obtained through a bioinformatic approach

Tecla Ciociola [1], Laura Giovati [1], Tiziano De Simone [1], Greta Bergamaschi [2], Alessandro Gori [2], Valerio Consalvi [3], Stefania Conti [1*] and Alberto Vitali [4]

1 Department of Medicine and Surgery, University of Parma, 43126 Parma, Italy; tecla.ciociola@unipr.it (T.C.); laura.giovati@unipr.it (L.G.); tiziano.desimone@unipr.it (T.D.S.); stefania.conti@unipr.it (S.C.)
2 National Research Council of Italy, Istituto di Scienze e Tecnologie Chimiche (SCITEC-CNR), 20131 Milan, Italy; greta.bergamaschi@scitec.cnr.it (G.B.); alessandro.gori@scitec.cnr.it (A.G.)
3 Dipartimento Scienze Biochimiche "A. Rossi Fanelli", Sapienza University of Rome, 00185 Roma, Italy. valerio.consalvi@uniroma1.it (V.C.)
4 National Research Council of Italy, Istituto di Scienze e Tecnologie Chimiche (SCITEC-CNR), 00168 Rome, Italy; alberto.vitali@scitec.cnr.it (A.V.)
* Correspondence: stefania.conti@unipr.it

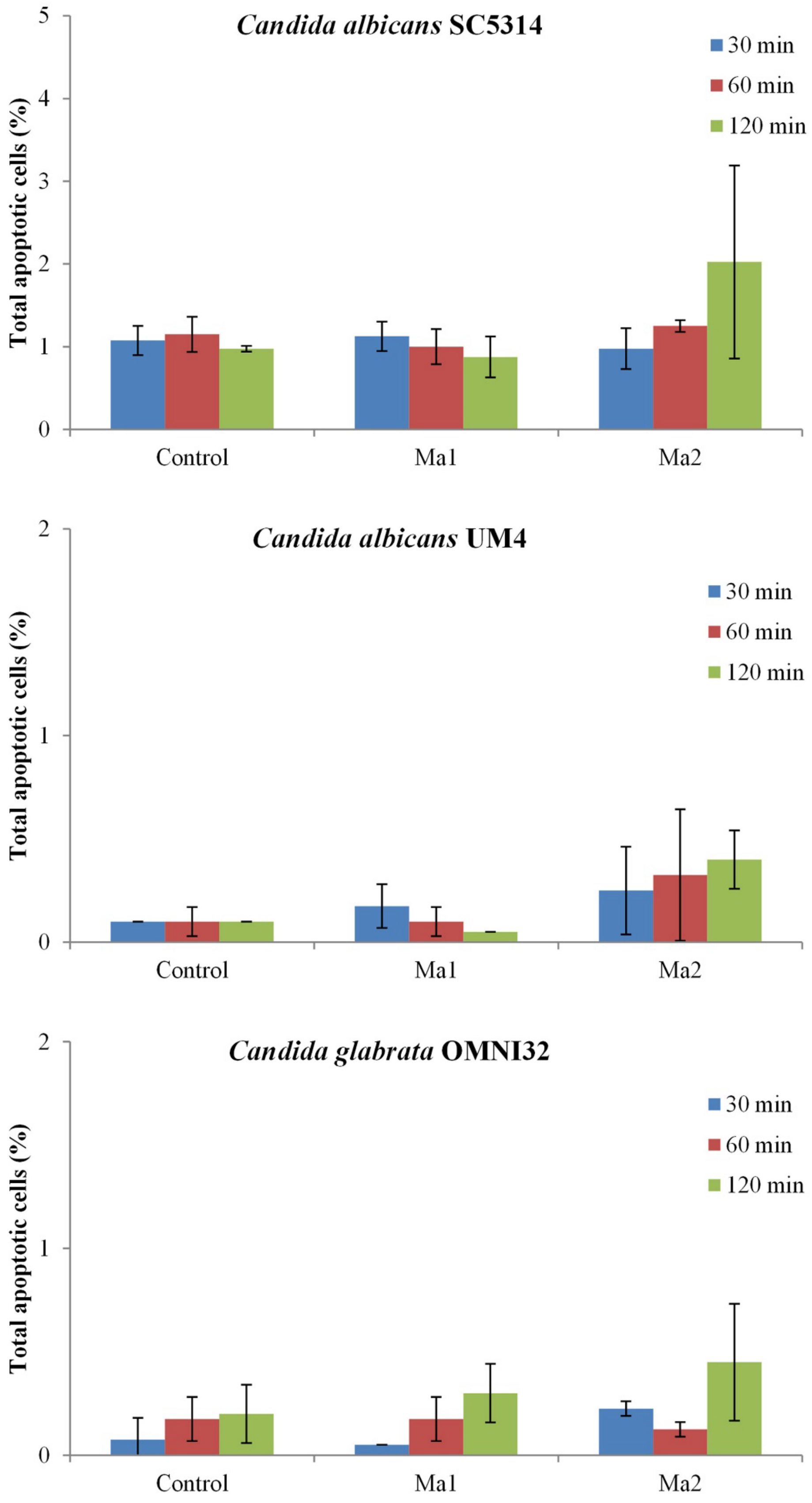

Figure S1. Apoptotic effects of treatment with Ma1 and Ma2 in *Candida* strains. Cells of *C. albicans* SC5314, *C. albicans* UM4, and *C. glabrata* OMNI32 were treated for 30, 60 and 120 min with peptides at their 2× EC_{50} value. Data, expressed as percentage of apoptotic cells on the total gated cells, represent the mean ± standard deviation from at least two independent experiments. Differences between peptide-treated groups and control (in the absence of peptides) were not statistically significant as assessed by Student’s t test.

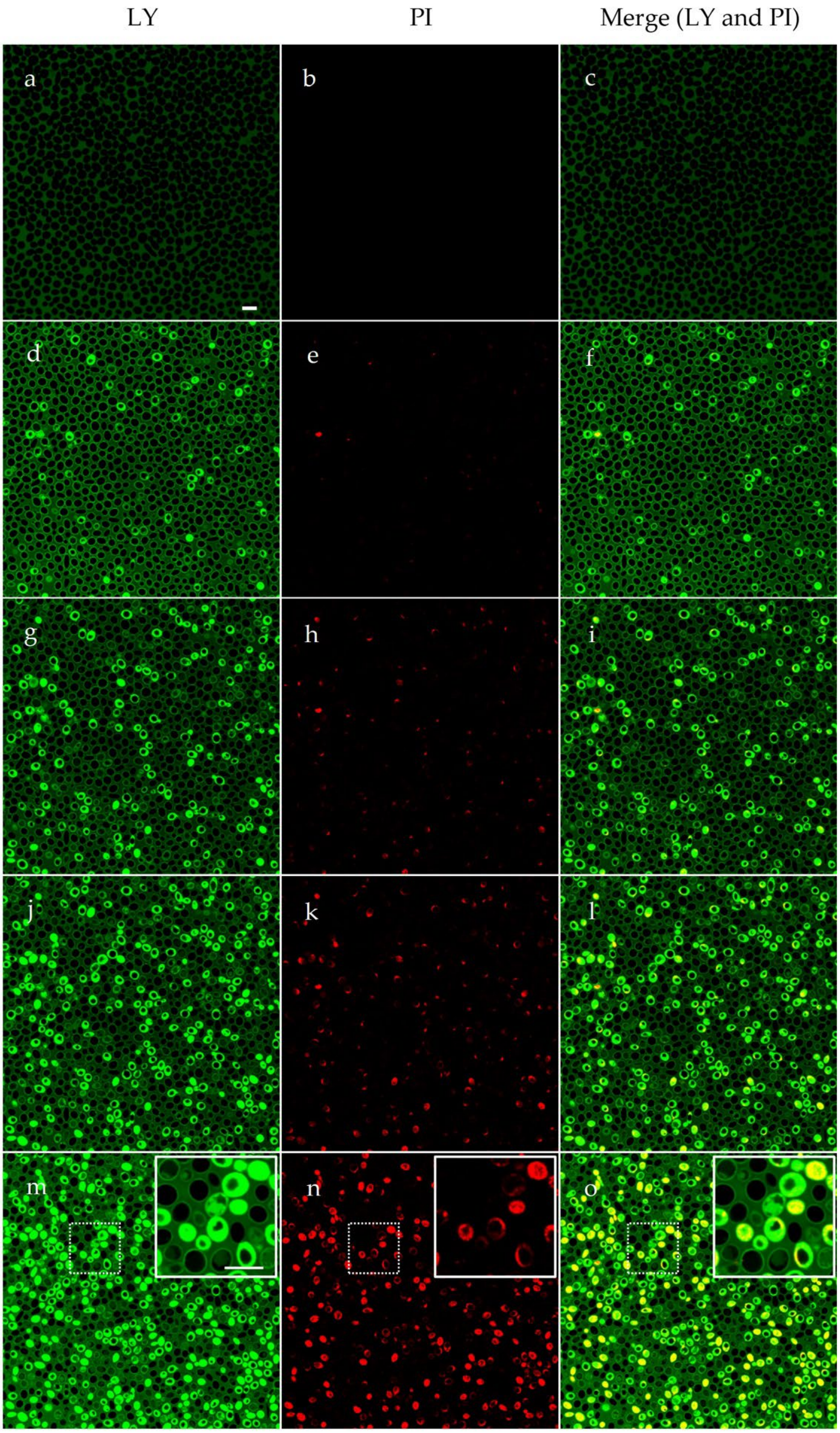

Figure S2. Confocal microscopy images of living *Candida albicans* UM4 cells treated with Ma1. (**a**-**c**): Yeast cells, pre-treated with propidium iodide (PI) and lucifer yellow (LY), were treated with Ma1. Images were taken after (**d**-**f**) 10 min, (**g**-**i**) 20 min, (**j**-**l**) 30 min, and (**m**-**o**) 60 min. The same field is shown, except for **a**-**c**. Over time, LY is increasingly evidenced inside yeast cells, with subsequent internalization of PI, suggesting an irreversible membrane permeabilization induced by the peptide, eventually resulting in cell death. Prior to internalization, LY is localized at the cell wall level. The inset in panels (**m**-**o**) shows a detail of the field highlighted by dotting. Bar, 10 μm.

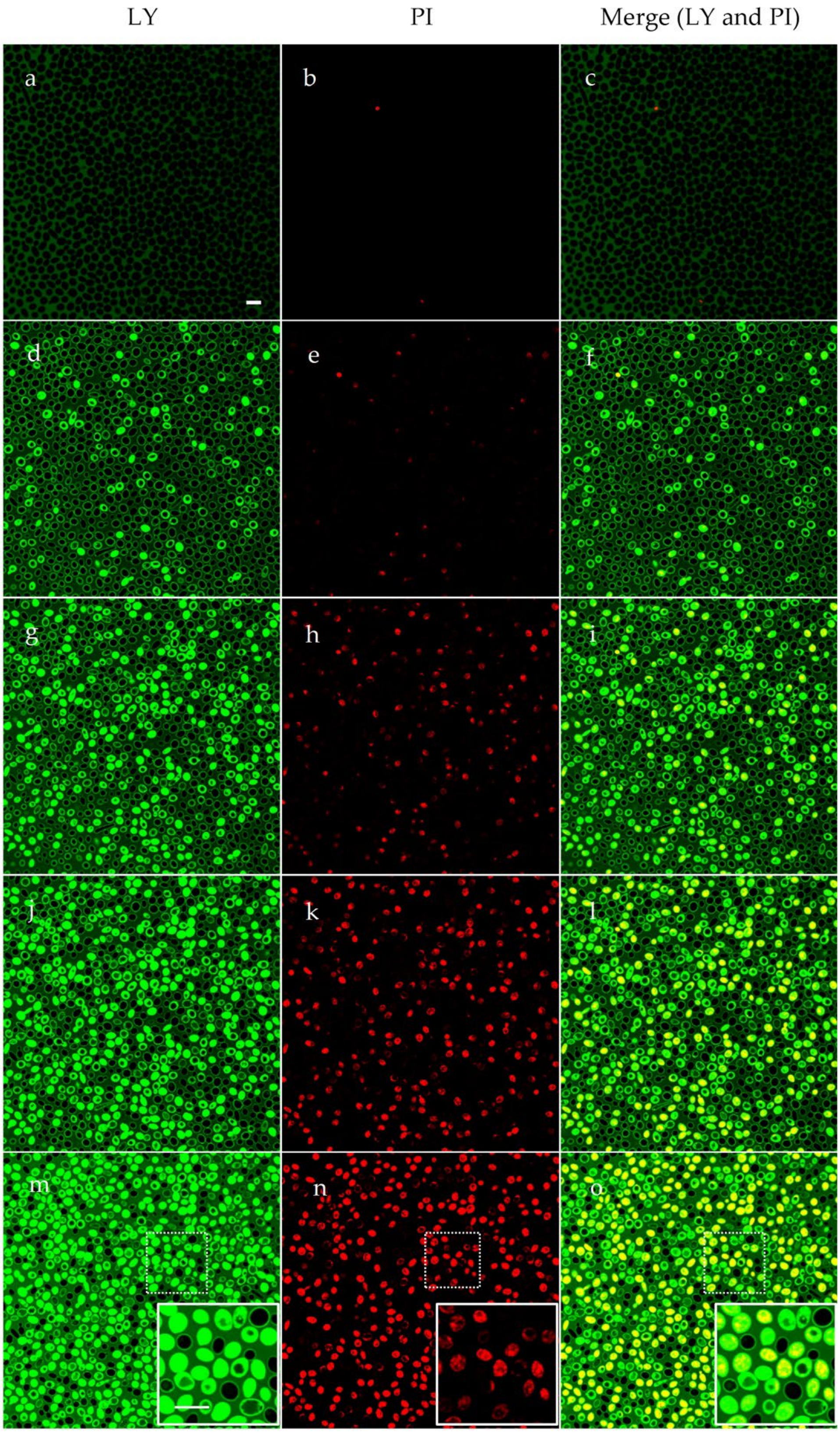

Figure S3. Confocal microscopy images of living *Candida albicans* UM4 treated with Ma2. (**a**-**c**): Yeast cells, pre-treated with propidium iodide (PI) and lucifer yellow (LY), were treated with Ma2. Images were taken after (**d**-**f**) 10 min, (**g**-**i**) 20 min, (**j**-**l**) 30 min, and (**m**-**o**) 60 min. The same field is shown. Over time, LY is increasingly evidenced inside yeast cells, with subsequent internalization of PI, suggesting an irreversible membrane permeabilization induced by the peptide, eventually resulting in cell death. Prior to internalization, LY is localized at the cell wall level. The inset in panels (**m**-**o**) shows a detail of the field highlighted by dotting. Bar, 10 μm.

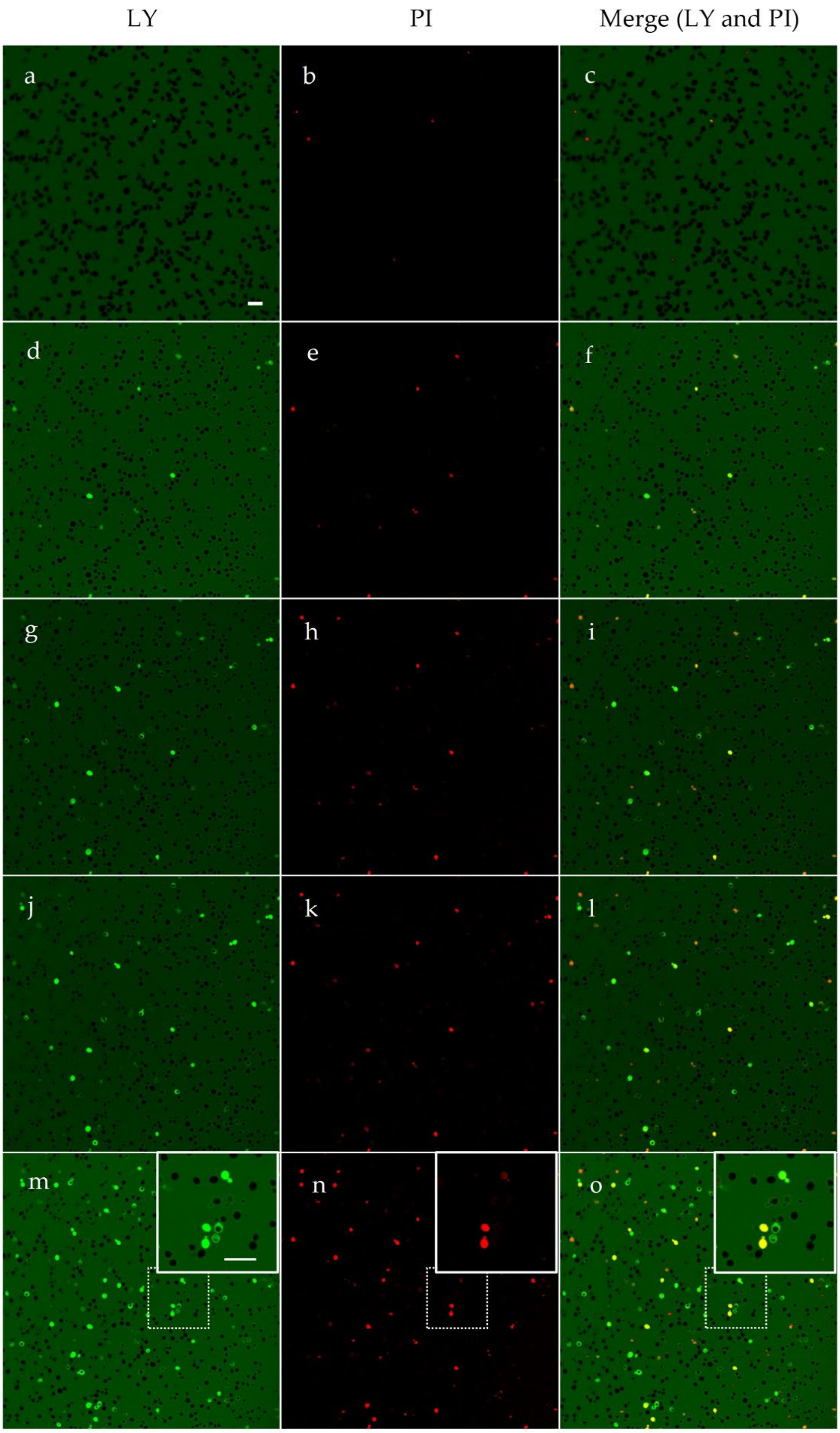

Figure S4. Confocal microscopy images of living *Candida glabrata* OMNI32 treated with Ma1. (**a**-**c**): Yeast cells, pre-treated with propidium iodide (PI) and lucifer yellow (LY), were treated with Ma1. Images were taken after (**d**-**f**) 10 min, (**g**-**i**) 20 min, (**j**-**l**) 30 min, and (**m**-**o**) 60 min. The same field is shown. Over time, LY is increasingly evidenced inside yeast cells, with subsequent internalization of PI, suggesting an irreversible membrane permeabilization induced by the peptide, eventually resulting in cell death. Prior to internalization, LY is localized at the cell wall level. The inset in panels (**m**-**o**) shows a detail of the field highlighted by dotting. Bar, 10 μm.

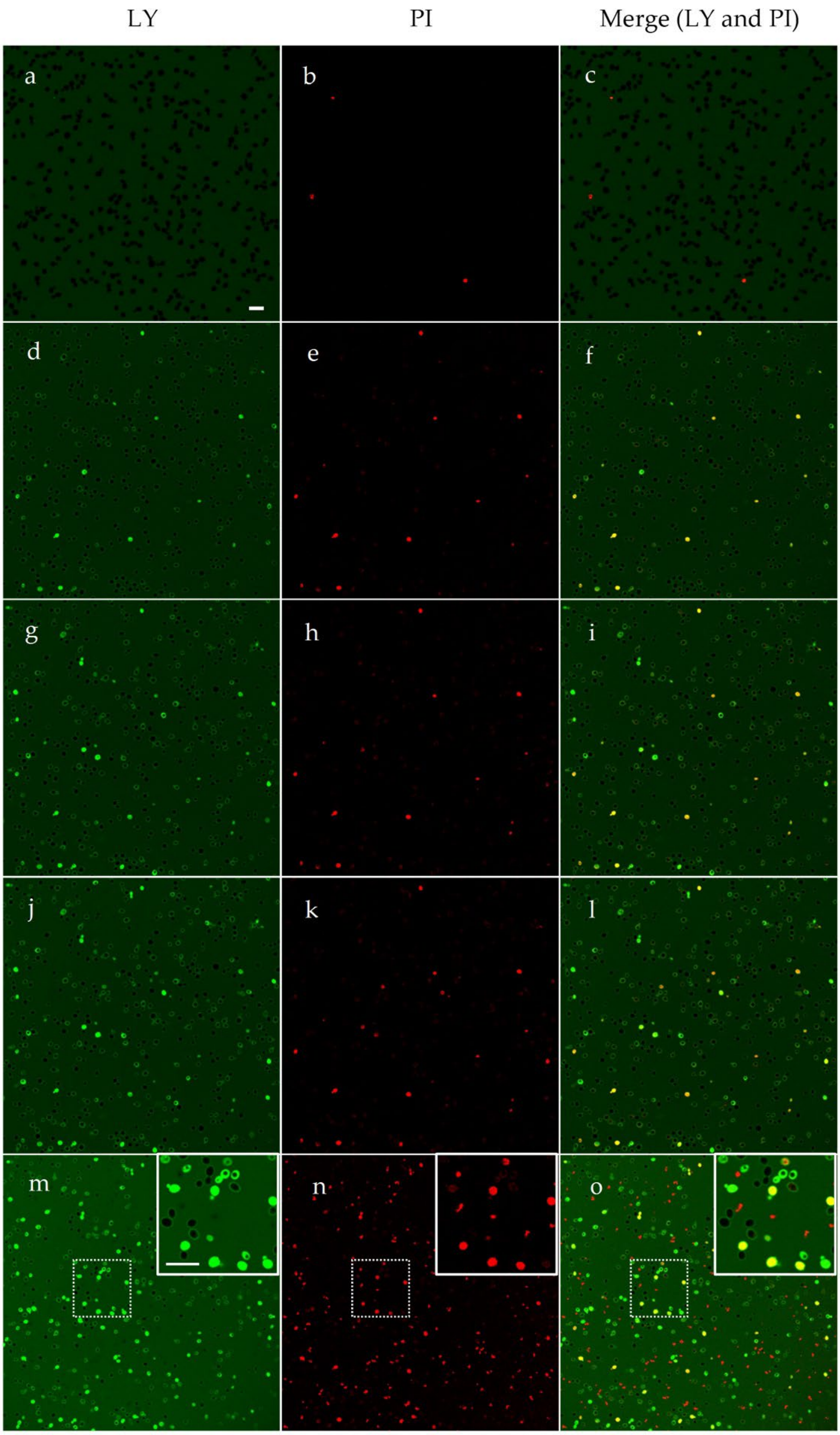

Figure S5. Confocal microscopy images of living *Candida glabrata* OMNI32 treated with Ma2. (**a**-**c**): Yeast cells, pre-treated with propidium iodide (PI) and lucifer yellow (LY), were treated with Ma2. Images were taken after (**d**-**f**) 10 min, (**g**-**i**) 20 min, (**j**-**l**) 30 min, and (**m**-**o**) 60 min. The same field is shown. Over time, LY is increasingly evidenced inside yeast cells, with subsequent internalization of PI, suggesting an irreversible membrane permeabilization induced by the peptide, eventually resulting in cell death. Prior to internalization, LY is localized at the cell wall level. The inset in panels (**m**-**o**) shows a detail of the field highlighted by dotting. Bar, 10 μm.

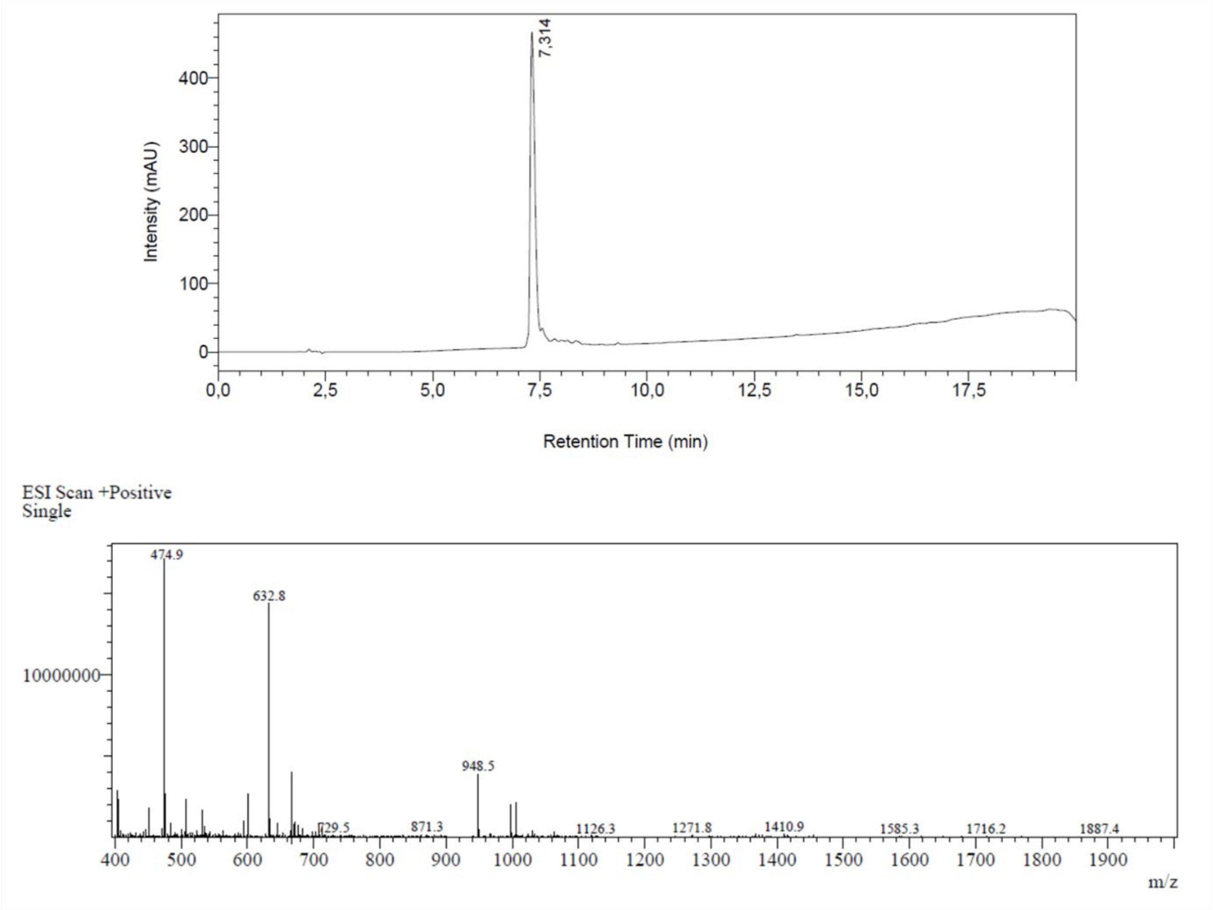

Figure S6. HPLC chromatogram (upper scheme) and mass spectrum (lower scheme) of Ma1 peptide.

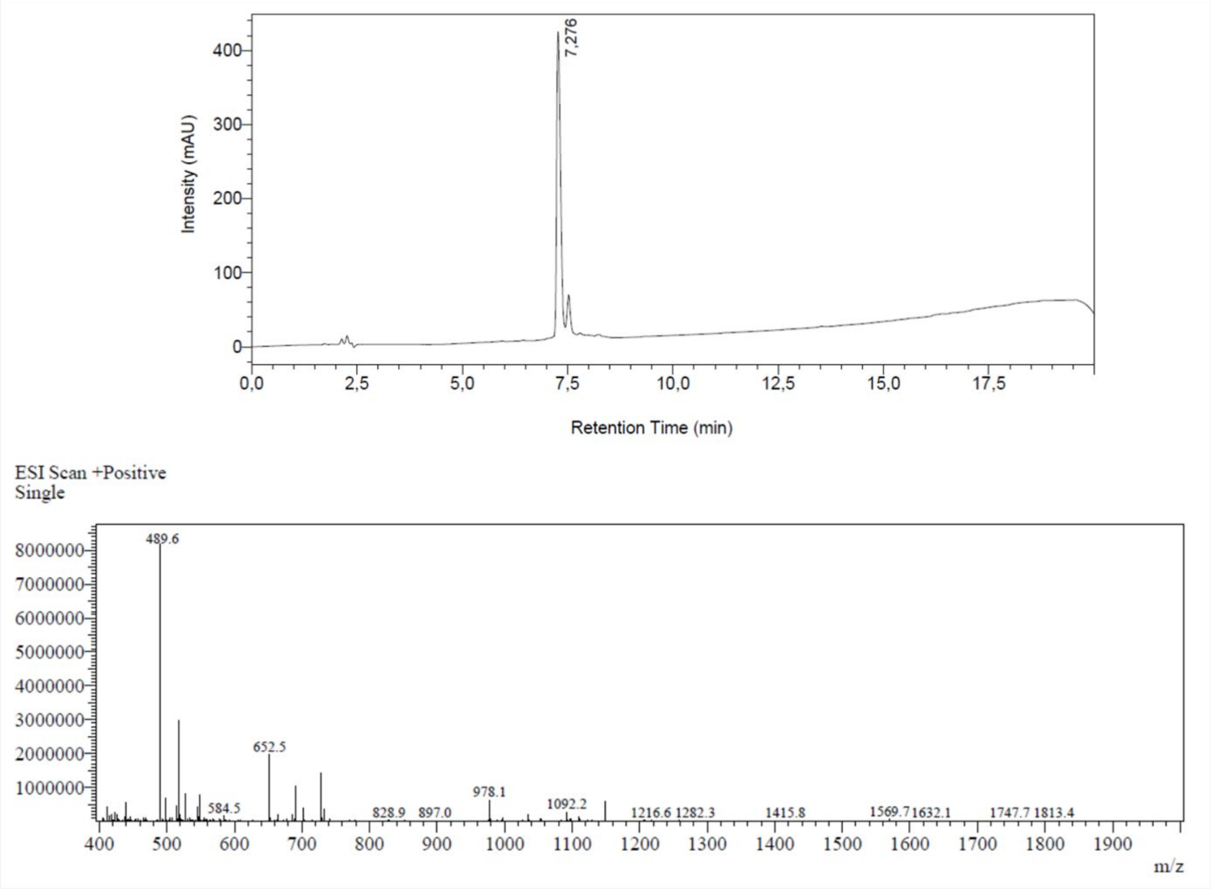

Figure S7. HPLC chromatogram (upper scheme) and mass spectrum (lower scheme) of Ma2 peptide.

Table S1. m/z values of obtained mass spectra of peptides Ma1 and Ma2.

CODE	SEQUENCE	CALCULATED MS	FOUND MS
**Ma1**	NH_2-PPKRPRRPRQPRLLQ-COOH	$[M+1]^{+}$= 1896.3; $[M+2]^{2+}$= 948.6; $[M+3]^{3+}$= 632.7; $[M+4]^{4+}$= 474.4;	$[M+2]^{2+}$= 948.5; $[M+3]^{3+}$= 632.8; $[M+4]^{4+}$= 474.9;
**Ma2**	NH_2-KRPRRPRQPRLLQRP-COOH	$[M+1]^{+}$= 1955.3; $[M+2]^{2+}$= 978.1; $[M+3]^{3+}$= 652.4; $[M+4]^{4+}$= 489.6;	$[M+2]^{2+}$= 978.1; $[M+3]^{3+}$= 652.5; $[M+4]^{4+}$= 489.6;
